# Telomere length in early childhood and its association with attention: a study in 4–6 year old children

**DOI:** 10.3389/fped.2024.1358272

**Published:** 2024-06-11

**Authors:** Hanne Croons, Dries S. Martens, Charlotte Vanderstukken, Hanne Sleurs, Leen Rasking, Martien Peusens, Eleni Renaers, Michelle Plusquin, Tim S. Nawrot

**Affiliations:** ^1^ Centre for Environmental Sciences, Hasselt University, Hasselt, Belgium; ^2^ Department of Public Health, Leuven University (KU Leuven), Leuven, Belgium

**Keywords:** cognition, telomere, attention, childhood, ENVIR*ON*AGE

## Abstract

Telomere length (TL), a marker of cellular aging, has been studied in adults with regard to its connection to cognitive function. However, little is known about the association between TL and cognitive development in children. This study investigated the interplay between TL and cognitive functioning in 283 Belgian children aged four to six years of the Environmental Influence on Aging in Early Life (ENVIR*ON*AGE) birth cohort. Child leukocyte TL was measured using qPCR, while cognitive functioning, including attention and memory, was assessed using the Cambridge Neuropsychological Test Automated Battery (CANTAB). Linear regression models were employed to examine the association between TL and cognitive outcomes, adjusting for potential confounders. We found an inverse association between TL and the spatial errors made during the Motor Screening task (*p* = 0.017), indicating a higher motor accuracy in children with longer telomeres. No significant associations were found between TL and other cognitive outcomes. Our results suggest a specific link between TL and motor accuracy but not with the other cognitive domains.

## 1 Introduction

Cognitive development, the mental processes involved in acquiring, storing, processing, and using information (1), is crucial for learning and adaptative skills, and often translates to higher academic accomplishments, more promising career opportunities, contributing to an overall better quality of life (2). It has been well established that environmental factors such as lifestyle, fitness, and nutrition, as well as genetic factors, contribute to cognitive functioning (3).

Recently, research has focused on the potential role of the biological underpinnings of cognition, including telomere biology (4). Telomeres are the protective caps at the ends of human chromosomes that play a crucial role in cellular aging (5). Telomere length (TL), reflects the replicative capacity of cells (6–8) and leukocyte telomere length (LTL) has been shown to be associated with cognitive function in several studies (9–13), but results are inconclusive (14–17) Previous research primarily focused on the association between TL and cognition in adults, particularly the elderly (4, 11, 13, 18), while the relationship between TL and neurodevelopmental outcomes in children has received little to no attention. To date, only one study has examined the association between TL and cognition in 209 children at the age of 5, and no association was observed (19).

Given that childhood is a crucial period for cognitive development, this study aims to investigate the association between leukocyte TL and cognitive functioning in children aged four to six years participating in the prospective Environmental Influence on Aging in Early Life (ENVIR*ON*AGE) birth cohort.

## 2 Methods

### 2.1 Study design and population

ENVIR*ON*AGE is an ongoing prospective birth cohort established in 2010 (Limburg, Belgium). Mother-child pairs, without planned cesarean section, and where the mother was able to fill out a Dutch language questionnaire, were recruited at birth at the East-Limburg Hospital (Genk, Belgium). When the child reached the age of 4 to 6 years old, they were invited for a follow-up visit at the university study center. Detailed information on the recruitment process at birth is provided elsewhere (20). Between October 2014 and October 2019, 439 mother-child pairs had a follow-up examination. The final ENVIR*ON*AGE population for our analysis consists of 283 children (Supplementary Figure S1). The ENVIR*ON*AGE study protocol is approved by the ethical committees of Hasselt University (Diepenbeek, Belgium, reference no. B371201216090 and B371201524537) and East-Limburg Hospital (Genk, Belgium). It has been carried out according to the Helsinki Declaration, and all mothers provided written informed consent.

### 2.2 Measures

Mothers filled out detailed questionnaires at the in order to obtain household and lifestyle information. Data on child sex, maternal and child age, maternal education and average hours the child sleeps were retrieved. Maternal education is coded “low” when mothers did not obtain a diploma, “middle” when obtained a high school diploma, and “high” when obtained a college or university degree. Based on the date of the examination day, a seasonal scale was calculated (Winter is from the 21st of December to the 20th of March, spring from the 21st of March to the 20th of June, summer from the 21st of June to the 20th of September, and autumn from the 21st of September to the 20th of December). Maternal health status regarding the occurrence of preeclampsia, hypertension, infectious diseases or diabetes during pregnancy was retrieved via medical records.

In addition to the detailed questionnaires, mothers also completed the Perceived Stress Scale (PSS) questionnaire to assess their stress levels, and the Strengths and Difficulties Questionnaire (SDQ) developed by Goodman (21) to evaluate the child's behavior using SDQ sub-scales (peer relationship, emotional, conduct, and hyperactivity) and a Total Difficulties Score. Lastly, body anthropometrics of the children, including height (to the nearest centimeter) and weight (to the nearest 0.1 kilograms), were measured by trained staff. Body mass index (BMI) was calculated as the ratio of weight over squared height.

### 2.3 Cognitive measurements

**Figure 1:**
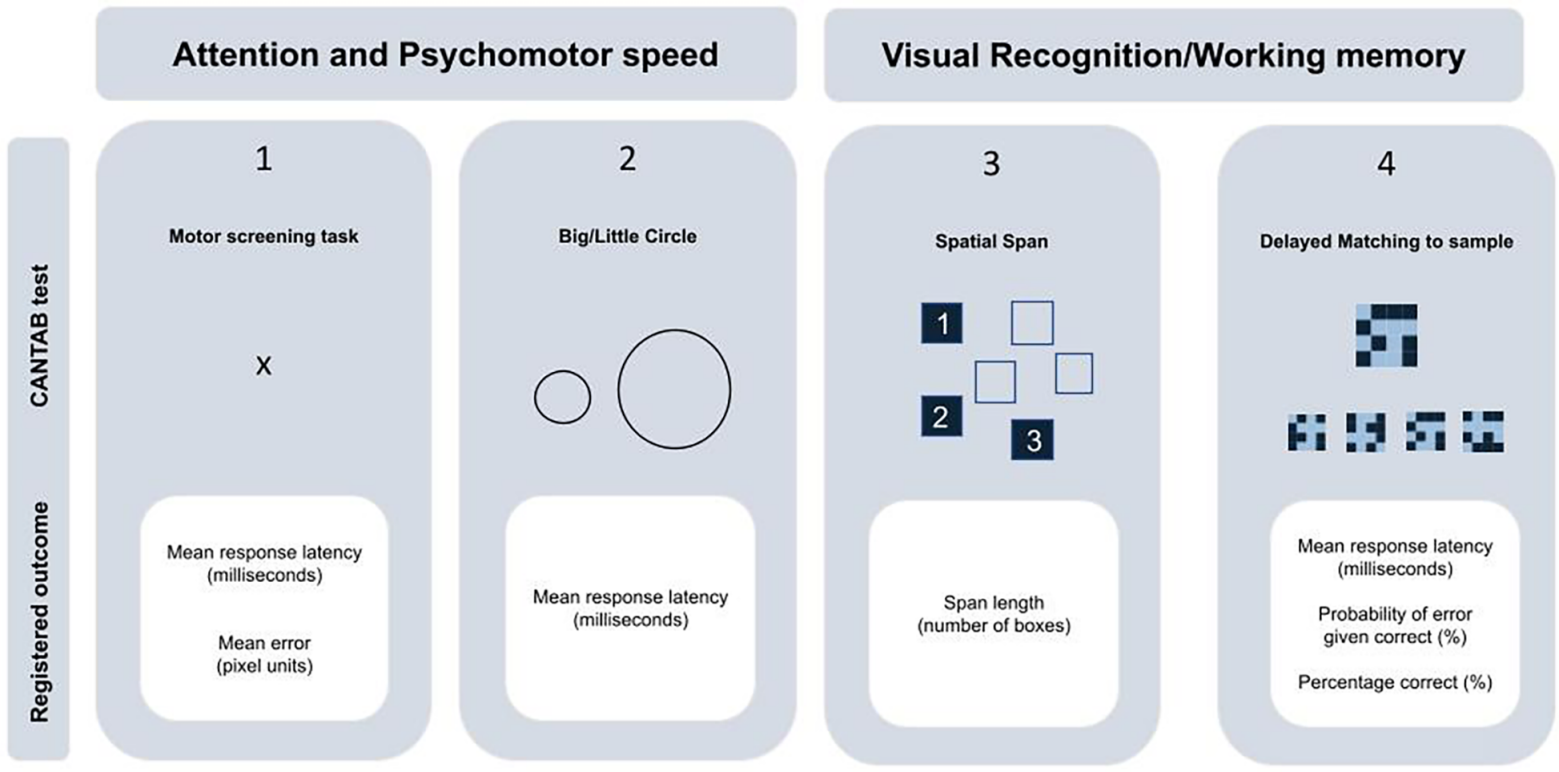
CANTAB testbattery overview.

The children's neurocognitive functioning was assessed using the Cambridge Neuropsychological Test Automated Battery (CANTAB) on a touch-screen tablet (CANTAB, Cognitive assessment software, 2019). The CANTAB has been shown to provide reliable measurements of executive functions in children as young as four years old (22). Trained examiners administered four tasks according to standardized instructions. Two of these tasks focused on attention and psychomotor speed: the Motor Screening Task and the Big/Little Circle Task, which assessed response accuracy (in pixels) and/or response latency (in milliseconds). The other two tasks assessed visual recognition and working memory. The Spatial Span task evaluated the maximum number of squares the child could remember in the correct sequence (span length). The Delayed Matching to Sample task measured response latency (in milliseconds), percentage correct (%), and error probability (%) (Figure 1). More detailed information on the CANTAB procedures can be found in Supplementary Text S1.

### 2.4 Relative average telomere length

Child blood was drawn and genomic DNA was isolated from the buffy coat of venous blood stored in EDTA tubes using the QIAamp® DNA mini kit (Qiagen GmbH, Hilden, Germany) following the manufacturer's instructions. DNA quantity and purity were assessed using the Nanodrop spectrophotometer (ND-1000; Isogen Life Science, De Meern, Netherlands) and the Quant-iT™ PicoGreen® dsDNA Assay Kit (Life Technologies, Foster City, CA, USA) using the Omega Fluostar plate reader (BMG LABTECH, Ortenberg, Germany). TL was measured using an adapted qPCR method described by Cawthon (23). Telomeres were measured in triplicate in a single batch. Telomere lengths were expressed as the ratio of telomere copy number to single-copy gene number (T/S) relative to the mean T/S ratio of the entire sample. Detailed sample collection procedures and TL-assay specifications, and reliability parameters are provided in Supplementary Text S2 and have been previously provided for the ENVIR*ON*AGE birth cohort (24).

### 2.5 Statistical analysis

Data was analyzed using JMP Pro 16.2.0 software. Participants' characteristics were presented as means (SD) or frequencies (%). All latency data, i.e., response latency of the Motor Screening Task, Big/Little Circle task, and Delayed Matching to Sample task were log-transformed (log10), to better comply with assumptions of model linearity. The association between the different cognitive outcomes and TL was assessed using multivariable-adjusted general linear models. The threshold for statistical significance was set at a 95% confidence level (*p *< 0.05). We adjusted our models for *a priori*-selected covariates that may be associated with both the dependent (cognitive outcomes) and independent variables (TL at 4–6 years old). In model 1, this includes the child's sex and age. In model 2, we further adjusted for the child's BMI, average sleep hours of sleep the child has on a typical day and night, maternal education, and the examination season. The normality of the residuals was visually evaluated by Q-Q plots. Estimates are presented as the difference in cognitive outcome for a 1-IQR increase in TL. For data that were log-transformed, we back-transformed the estimates and expressed them as a percentage difference.

To assess the robustness of our findings, we performed several sensitivity analyses on the computerized measures of cognitive function. First, we excluded children who showed any signs of disinterest or were distracted while taking the cognitive tests to account for possible response errors. Second, we adjusted our models for the child's behavior over the past six months by using four sub-scales of the SDQ questionnaire: the peer relationship and emotional subscales, and the conduct and hyperactivity subscales, each scored as normal vs. not normal. Further we additionally adjusted our models for PSS scores and maternal health conditions during pregnancy like diabetes, preeclampsia, hypertension or infections. Lastly, we stratified by sex.

## 3 Results

### 3.1 ENVIR*ON*AGE study population

Characteristics of the 283 participants are provided in Table 1. Children were on average (SD) 4.57 (0.41) years old, and approximately half of them were boys (51.9%). Most participants were of European ethnicity (94.0%), with an average BMI of 16.05 (1.26) kg/m^2^. The season in which the examinations took place was for 64 (22.6%) participants during autumn, 88 (31.1%) participants during spring, 71 (25.1%) participants during summer, and 60 participants (21.2%) during winter. The relative telomere length ranged from 0.64 to 1.47. Accompanying mothers were on average 30.1(4.4) years old, and the majority had a college education or higher (62.9%).

**Table 1:** Characteristics of the 283 ENVIR*ON*AGE participants.

Characteristics	Mean (SD) or frequency (%)
Child	
Age at the visit, years	4.57 (0.41)
Sex	
Male	144 (51.9%)
Female	139 (49.1%)
Ethnicity	
European	266 (94.0%)
Non-European	17 (6.0%)
BMI Child	16.05 (1.26)
Sleep hours	10.88 (1.04)
Season of examinations	
Autumn	64 (22.6%)
Spring	88 (31.1%)
Summer	71 (25.1%)
Winter	60 (21.2%)
Telomere Length (range)	0.64–1.47
SDQ score^a^	
Emotional	211 (82%)
Hyperactivity	198 (77%)
Conduct	201 (78%)
Peer problem	218 (84%)
Prosocial	244 (95%)
Total	222 (86%)
Mother	
Age	30.1 (4.4)
Education	
Low	27 (9.5%)
Middle	78 (27.6%)
High	178 (62.9%)
PSS score	14.52 (6.06)
Health conditions during pregnancy	
Diabetes	8 (2.8%)
Preeclampsia	9 (3.2%)
Hypertension	3 (1,1%)
Infectious diseases	4 (1,4%)

^a^ SDQ scores based on 256 participants, frequency represents the participants with normal SDQ scores.

SDQ data was derived from 256 participants and categorized results into “normal” and “not normal” for each subscore. For the majority of participants (82%), the emotional symptom score falls within the “normal” range. The same is true for the hyperactivity score, where 77% exhibited behaviors classified as “normal”, signifying a typical level of hyperactivity. Further, 78% of participants demonstrated “normal” conduct, indicating a standard range of behavioral conduct. Within the domain of peer relationships, 84% of participants showcased “normal” patterns, underscoring healthy social interactions. Moreover, 95% of participants exhibited “normal” prosocial behaviors, meaning they have a high degree of positive engagement and cooperation with others. The combination of these individual sub-scales resulted in a comprehensive assessment of participants’ behavioral characteristics, quantified by the Total Difficulties Score. 86% of participants received a “normal” classification according to this measure.

### 3.2 Cognitive performance outcomes

The outcomes for the four CANTAB tasks are summarized in Table 2. The median (p25–p75) response latency of the Motor Screening task referring to the average time the child needed to touch the target successfully, was 964.4 (803–1148.9) milliseconds. The median error, measured as pixel units from the target center made during the same task, was 14.3 (12.2–16.0). In the Big/Little circle, the response latency was similarly calculated as the average time to select the correct circle and was observed to be 1092.4 (965.7–1228.6) milliseconds. During the Spatial Span task, participants demonstrated an ability to accurately reproduce a median of 3 (2–3) squares in the correct sequence. Finally, in the Delayed Matching to Sample task, participants needed a median time of 4133.1 (3282.8–5134.0) milliseconds to accurately select the correct pattern on the first try for all presented sample patterns. The percentage of trials answered correctly on the first try was 45.0 (35.0–55.0)% and the probability of error given a previous correct answer was 0.6% (0.4–0.7).

**Table 2:** Characteristics CANTAB outcomes for the 283 ENVIR*ON*AGE participants.

	Min	P25	P50	P75	Max
Motor Screening Task					
Response Latency, ms	535.1	803	946.4	1148.9	1946.1
Error, pixel units	6.65	12.2	14.3	16.0	21.5
Big/Little Circle Task					
Response Latency, ms	683	965.7	1092.4	1228.6	2719.9
Spatial Span Task					
Span length	0	2	3	3	5
Delayed Matching to Sample Task					
Response Latency, ms	1376.6	3286.8	4142.9	5127.7	13994.7
Percentage correct, %	10	35	45	55	85
Probability of error given correct, %	0.1	0.4	0.6	0.7	1.0

### 3.3 TL and cognitive performance outcomes

**Figure 2:**
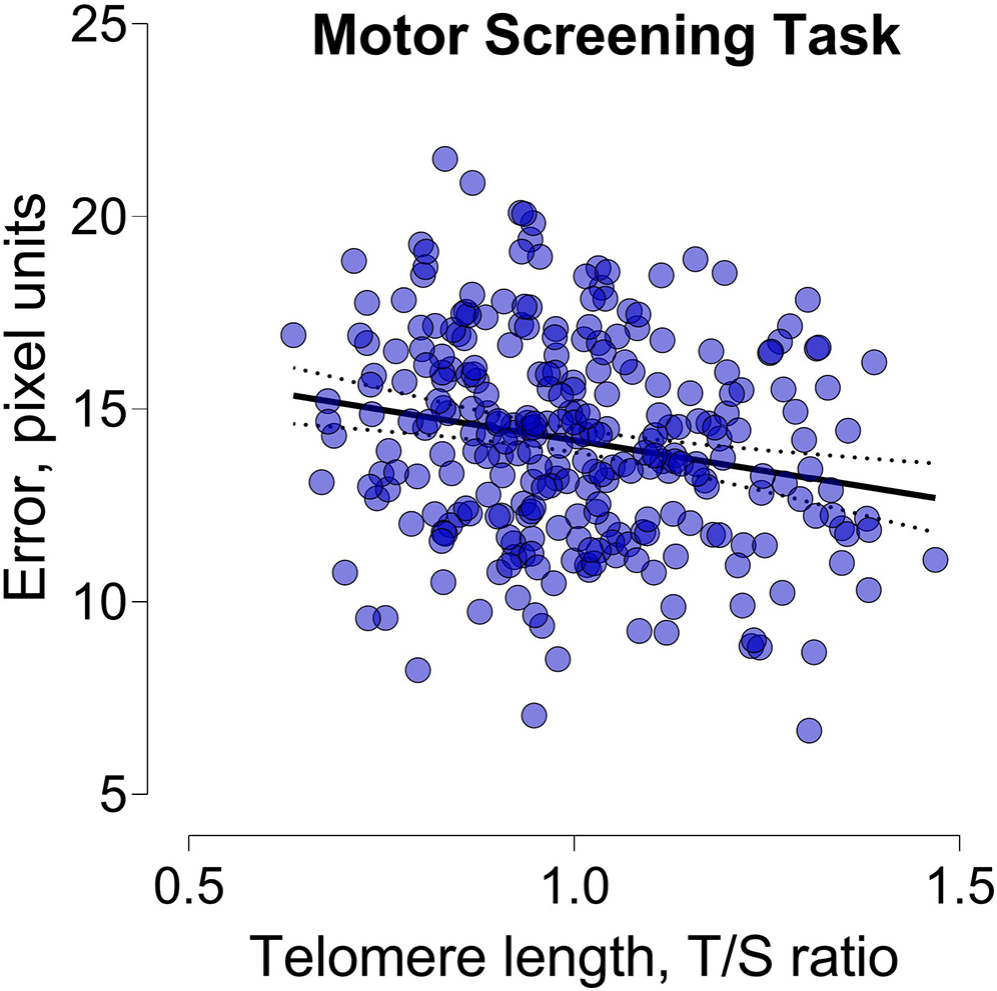
Scatterplot illustrating the relationship between telomere length and the error made during the motor screening task.

In unadjusted analysis, we only observed a negative association between TL and the error made during the Motor screening task (Figure 2). A 1-IQR increase in TL was associated with a 0.58-pixel unit lower pixel error (95% CI: −1.04 to −0.12; *p *= 0.014). After adjustment for child sex and age the estimates were comparable and a 1-IQR increment in TL was associated with a 0.57-pixel unit decrease. In fully adjusted models this association remained significant (95% CI: −1.04 to −0.10; *p *= 0.017). No other cognitive outcomes related to attention and memory were found to be associated with childhood TL (Table 3).

**Table 3:** Association between child TL and cognition in children aged 4 to 6 years.

	Unadjusted model		Model 1		Model 2	
	Estimate (95% CI)	*p*	Estimate (95% CI)	*p*	Estimate (95% CI)	*p*
Motor screening task						
Response Latency, ms	−0.28 (−4.28, 3.88)	0.89	0.30 (−3.41, 4.16)	0.88	0.42 (−3.33, 4.32)	0.83
Error, pixel units	−0.58 (−1.04, −0.12)	**0** **.** **014**	−0.56 (−1.02, −0.10)	**0** **.** **018**	−0.57 (−1.04, −0.10)	**0** **.** **017**
Big/little circle task						
Response Latency, ms	−1.31 (−4.57, 2.05)	0.44	−0.14 (−3.20, 2.99)	0.92	−0.20 (−3.25, 2.95)	0.90
Spatial span task						
Span length	0.054 (−0.16, 0.27)	0.63	0.017 (−0.19, 0.23)	0.88	−0.01 (−0.22, 0.20)	0.94
Delayed matching to sample task						
Response Latency, ms	3.6 (−2.58, 10.17)	0.26	4.08 (−2.23, 1079)	0.21	4.10 (−2.25, 10.85)	0.21
Percentage correct, %	0.72 (−1.94, 3.39)	0.59	0.04 (−2.53, 2.60)	0.98	0.004 (−2.60, 2.60)	1.00
Probability of error given correct, %	−0.002 (−0.04,0.03)	0.91	0.004 (−0.03,0.04)	0.84	0.006 (−0.03,0.04)	0.72

Estimates are presented as a difference in cognitive outcome for a 1-IQR increase in TL. Latency data is expressed as the percentage difference in cognitive outcome for a 1-IQR increase in TL.

Model 1 adjusted for sex and age.

Model 2 adjusted according to model 1 (sex, age) with additional adjustment for child BMI, average sleep hours, diploma of the mother, and the season of the examinations. Statistically significant values are highlighted in bold.

### 3.4 Sensitivity analysis

Results of the sensitivity analyses are shown in Supplementary Table S1. The association between TL and the error made during the Motor screening task remained robust after the exclusion of children who were disinterested or unfocused during the tests, and for additional adjustment for SDQ results, PSS results and maternal health conditions during pregnancy. After stratification for child sex, a 1-IQR increment in TL was associated with a 0.86 decrease in this error in boys and a and 0.49 decrease in this error in girls, and no significant interaction was observed (p-interaction = 0.93).

## 4 Discussion

In this study, we evaluated the association between leukocyte TL and cognitive performance in 4–6-year-old children specifically focusing on two domains of cognition: memory and attention. However, we found no evidence of a strong association. Nevertheless, we did observe that children with longer telomeres were more accurate in the Motor Screening Task, as shown by a decrease of 0.58-pixel unit distance from the target center, for a 1-IQR increase in TL. These findings were confirmed after adjustment for potential confounding factors.

Our study adds to the limited research examining the association between cognition and TL in children. In a study by Feiler et al. (19), no association was observed between leukocyte TL and neurodevelopmental outcomes in 209 5-year-old children, despite using a finger-tapping test similar to our motor screening task. It is important to note that the latter focused solely on latency and did not consider the distance between the object and the point of contact on the screen (19). In addition, several studies have found an association between TL and ADHD in children (7, 25, 26).

While early-life biological aging differences may not yet manifest as significant cognitive performance disparities in children, there is a growing recognition of the association between TL and various cognitive outcomes later in life. For instance, Valdes et al. found significant correlations between TL and CANTAB outcomes in a cross-sectional survey including 382 women aged 19 to 78 years (12). Similarly, TL has been linked to Modified Mini-Mental State Exam (MMSE) scores, a test widely used to assess global cognitive function, in 976 older men (11) and 17,052 adults (10). Additionally, associations have been found between TL and the composite score from six cognitive tests in 2,092 nurses (9). TL has also been linked to information processing speed, visual-spatial function, and memory, albeit to a lesser extent in the attention and executive domains (27).

Longitudinal investigations are crucial to capture the cumulative effects of early-life biological aging differences on cognition. Martin-Ruiz et al. found that longer telomeres at baseline was associated with less reduction in 2-year Modified Mini-Mental State Exam change scores in stroke survivors (28). Furthermore, elderly with longer TL exhibited better baseline attention and psychomotor speed and less decline in global cognitive functioning over seven years relative to elders with shorter TL (13). Other longitudinal studies have, however, failed to observe such effects in other populations (16, 17). Similarly, some cross-sectional studies did not observe significant associations of TL with general cognitive decline (14, 15) or specific cognitive domains (10, 14). Discrepancies in findings across these studies may be attributed to factors such as sample size, participant characteristics, TL assessment methods, and the types of cognitive measures used.

Children's brains demonstrate remarkable neuroplasticity, enabling rapid learning and adaptation. New neural connections readily emerge, fostering cognitive development. Consequently, the mechanisms linking telomere length to cognition may diverge between children and the elderly. At present, we can only speculate about possible mechanisms when investigating the link between TL and cognition. Possible mechanisms include: (i) oxidative stress and inflammation, which are commonly associated with aging, TL, and cognitive performance (29, 30). It is worth noting that brain tissue is particularly susceptible to oxidative stress due to its moderate levels of antioxidants, despite its high energy demands (31). Another hypothesized mechanism suggests (ii) a shared genetic basis for both the shortening of telomere length and the onset of cognitive decline (27).

 Studies examining the association between TL and gray matter atrophy concluded that shorter telomeres are associated with gray matter atrophy, mainly in the subcortical/limbic regions (32) which are found to be associated with children's cognitive abilities (33). Gampawar et al. stated that about half of the effect of TL on cognition in adults was mediated by the brain parenchymal fraction, which is the ratio of brain parenchymal (functional) volume to total brain volume (34). Better connectivity has also been observed with longer telomeres (35) and is a significant predictor of better performances in the attention/speed domain (36). An extensive meta-analysis on the role of telomere length in brain aging so far confirmed these results and showed that longer telomeres are indeed beneficial to both brain structure and cognition during aging (6).

Our study is among the first to investigate the relationship between TL and the cognitive functioning of children as young as four. Our study has several important strengths. First, we assessed neurocognition in children during the most critical period of brain development (37). Sensitivity analyses showed minimal changes to the observed associations, indicating robust and consistent results. We accounted for a potential lack of motivation causing inaccurate response validity in our sensitivity analysis. Furthermore, we also used leukocyte TL as a proxy for TL, since it was shown to correlate well with TL across different tissues within individuals (38). Nevertheless, we acknowledge the following limitations. First, this is a cross-sectional study, so we were unable to evaluate rates of change in these variables, which might be a more informative metric than a single measurement. Second, cognitive measurements are subject to random measurement error, such that we expect a certain amount of non-differential misclassification in our outcome, which might have attenuated our results. Even though results were measured in children during the most critical period of brain development, the pathology underlying cognitive impairments appears to begin decades prior to the onset of detectable symptoms, and thus our measurement of cognition might be quite early to be able to predict more obvious cognitive decline (9). In addition to that, our study population of 283 children might be relatively small to detect this possible decline, particularly if the effects are subtle and less pronounced in this early life stage as compared to later life cognitive effects. Last, we only found an association in one of the subdomains of attention. No associations were found for the other attention-related domains.

In conclusion, we found that LTL is only associated with higher motor accuracy in children aged 4 to 6 years. Currently, we have no evidence that memory or other domains of attention are associated with TL. Future studies with a larger sample size, prospective design, and other relevant biological markers (e.g., oxidative stress) are needed to clarify the role of TL in cognitive performances in children.

## Data Availability

The data analyzed in this study is subject to the following licenses/restrictions: Requests to access these datasets should be directed to hanne.croons@uhasselt.be.

## References

[1] Thompson R Smith RB Karim YB Shen C Drummond K Teng C Air pollution and human cognition: a systematic review and meta-analysis. Sci Total Environ. (2023) 859:160234. 10.1016/j.scitotenv.2022.160234 36427724

[2] Lima RA Soares FC van Poppel M Savinainen S Mäntyselkä A Haapala EA Determinants of cognitive performance in children and adolescents: a populational longitudinal study. Int J Environ Res Public Health. (2022) 19:15. 10.3390/ijerph19158955 PMC9331797 35897325

[3] Tucker-Drob EM Briley DA Harden KP. Genetic and environmental influences on cognition across development and context. Curr Dir Psychol Sci. (2013) 22(5):349–55. 10.1177/0963721413485087 24799770 PMC4006996

[4] Diukov Y Bachinskaya N Dzobak A Kholin V Kyriachenko Y Barsukov O Association of telomere length with cognitive impairment. J Mol Neurosci. (2023) 73(6):448–55. 10.1007/S12031-023-02130-1 37278929

[5] Shay JW. Telomeres and aging. Curr Opin Cell Biol. (2018) 52:1–7. 10.1016/j.ceb.2017.12.001 29253739

[6] Gampawar P Schmidt R Schmidt H. Telomere length and brain aging: a systematic review and meta-analysis. Ageing Res Rev. (2022) 80:101679. 10.1016/J.ARR.2022.101679 35777725

[7] Pham C Vryer R O’hely M Mansell T Burgner D Collier F Shortened infant telomere length is associated with attention deficit/hyperactivity disorder symptoms in children at age two years: a birth cohort study. Int J Mol Sci. (2022) 23(9):4601. 10.3390/IJMS23094601/S1 35562991 PMC9104809

[8] Wikgren M Karlsson T Söderlund H Nordin A Roos G Nilsson LG Shorter telomere length is linked to brain atrophy and white matter hyperintensities. Age Ageing. (2014) 43(2):212–7. 10.1093/ageing/aft172 24231584

[9] Devore EE Prescott J De Vivo I Grodstein F. Relative telomere length and cognitive decline in the Nurses’ health study. Neurosci Lett. (2011) 492(1):15–8. 10.1016/j.neulet.2011.01.041 21295115 PMC3306217

[10] Hägg S Zhan Y Karlsson R Gerritsen L Ploner A Van Der Lee SJ Short telomere length is associated with impaired cognitive performance in European ancestry cohorts. Transl Psychiatry. (2017) 7:4. 10.1038/tp.2017.73 PMC5416710 28418400

[11] Ma SL Lau ESS Suen EWC Lam LCW Leung PC Woo J Telomere length and cognitive function in southern Chinese community-dwelling male elders. Age Ageing. (2013) 42(4):450–5. 10.1093/ageing/aft036 23519133

[12] Valdes AM Deary IJ Gardner J Kimura M Lu X Spector TD Leukocyte telomere length is associated with cognitive performance in healthy women. Neurobiol Aging. (2010) 31(6):986–92. 10.1016/j.neurobiolaging.2008.07.012 18718693 PMC2876308

[13] Yaffe K Lindquist K Kluse M Cawthon R Harris T Hsueh WC Telomere length and cognitive function in community-dwelling elders: findings from the health ABC study. Neurobiol Aging. (2011) 32(11):2055–60. 10.1016/j.neurobiolaging.2009.12.006 20031273 PMC2916948

[14] Harris SE Deary IJ MacIntyre A Lamb KJ Radhakrishnan K Starr JM The association between telomere length, physical health, cognitive ageing, and mortality in non-demented older people. Neurosci Lett. (2006) 406(3):260–4. 10.1016/j.neulet.2006.07.055 16919874

[15] Kaja R Reyes SM Rossetti HC Brown ES. Association between telomere length and cognitive ability in a community-based sample. Neurobiol Aging. (2019) 75:51–3. 10.1016/j.neurobiolaging.2018.11.006 30544048

[16] Mather KA Jorm AF Anstey KJ Milburn PJ Easteal S Christensen H. Cognitive performance and leukocyte telomere length in two narrow age-range cohorts: a population study. BMC Geriatr. (2010) 10:62. 10.1186/1471-2318-10-62 20843367 PMC2949672

[17] Zhan Y Clements MS Roberts RO Vassilaki M Druliner BR Boardman LA Association of telomere length with general cognitive trajectories: a meta-analysis of four prospective cohort studies. Neurobiol Aging. (2018) 69:111–6. 10.1016/j.neurobiolaging.2018.05.004 29870951 PMC6064381

[18] Han MH Lee EH Park HH Choi SH Koh SH. Relationship between telomere shortening and early subjective depressive symptoms and cognitive complaints in older adults. Aging. (2023) 15(4):914–31. 10.18632/AGING.204533 36805537 PMC10008503

[19] Feiler MO Patel D Li H Meacham PJ Watson GE Shamlaye C The association between early-life relative telomere length and childhood neurodevelopment. Neurotoxicology. (2018) 65:22–7. 10.1016/j.neuro.2018.01.005 29360532 PMC5857245

[20] Janssen BG Madhloum N Gyselaers W Bijnens E Clemente DB Cox B Cohort profile: the ENVIRonmental influence on early AGEing (ENVIR*ON*AGE): a birth cohort study. Int J Epidemiol. (2017) 46(5):1386–1387M. 10.1093/ije/dyw269 28089960

[21] Goodman R. Psychometric properties of the strengths and difficulties questionnaire. J Am Acad Child Adolesc Psychiatry. (2001) 40(11):1337–45. 10.1097/00004583-200111000-00015 11699809

[22] Luciana M. Practitioner review: computerized assessment of neuropsychological function in children: clinical and research applications of the Cambridge Neuropsychological Testing Automated Battery (CANTAB). J Child Psychol Psychiatry Allied Discip. (2003) 44(5):649–63. 10.1111/1469-7610.00152 12831110

[23] Cawthon RM. Telomere length measurement by a novel monochrome multiplex quantitative PCR method. Nucleic Acids Res. (2009) 37:3. 10.1093/nar/gkn1027 PMC2647324 19129229

[24] Martens DS Van Der Stukken C Derom C Thiery E Bijnens EM Nawrot TS. Newborn telomere length predicts later life telomere length: tracking telomere length from birth to child- and adulthood. EBioMedicine. (2021) 63:103164. 10.1016/j.ebiom.2020.103164 33422989 PMC7808927

[25] Costa DDS Rosa DVF Barros AGA Romano-Silva MA Malloy-Diniz LF Mattos P Telomere length is highly inherited and associated with hyperactivity-impulsivity in children with attention deficit/hyperactivity disorder. Front Mol Neurosci. (2015) 8:28. 10.3389/fnmol.2015.00028 26217174 PMC4498098

[26] Wojcicki JM Heyman MB Elwan D Shiboski S Lin J Blackburn E Telomere length is associated with oppositional defiant behavior and maternal clinical depression in Latino preschool children. Transl Psychiatry. (2015) 5:e581. 10.1038/tp.2015.71 26080316 PMC4490282

[27] Cohen-Manheim I Doniger GM Sinnreich R Simon ES Pinchas R Aviv A Increased attrition of leukocyte telomere length in young adults is associated with poorer cognitive function in midlife. Eur J Epidemiol. (2016) 31(2):147–57. 10.1007/s10654-015-0051-4 26076919 PMC4819924

[28] Martin-Ruiz C Dickinson HO Keys B Rowan E Kenny RA Von Zglinicki T. Telomere length predicts poststroke mortality, dementia, and cognitive decline. Ann Neurol. (2006) 60(2):174–80. 10.1002/ana.20869 16685698

[29] Barnes RP Fouquerel E Opresko PL. The impact of oxidative DNA damage and stress on telomere homeostasis. Mech Ageing Dev. (2019) 177:37–45. 10.1016/j.mad.2018.03.013 29604323 PMC6162185

[30] Kandlur A Satyamoorthy K Gangadharan G. Oxidative stress in cognitive and epigenetic aging: a retrospective glance. Front Mol Neurosci. (2020) 13:41. 10.3389/fnmol.2020.00041 32256315 PMC7093495

[31] Öğütlü H Esin İS Erdem HB Tatar A Dursun OB. Mitochondrial DNA copy number is associated with attention deficit hyperactivity disorder. Psychiatr Danub. (2020) 32(2):168–75. 10.24869/psyd.2020.168 32796781

[32] Yu J Kanchi MM Rawtaer I Feng L Kumar AP Kua EH The functional and structural connectomes of telomere length and their association with cognition in mild cognitive impairment. Cortex. (2020) 132:29–40. 10.1016/j.cortex.2020.08.006 32919107

[33] Pangelinan MM Zhang G VanMeter JW Clark JE Hatfield BD Haufler AJ. Beyond age and gender: relationships between cortical and subcortical brain volume and cognitive-motor abilities in school-age children. Neuroimage. (2011) 54(4):3093–100. 10.1016/j.neuroimage.2010.11.021 21078402 PMC3020257

[34] Gampawar P Schmidt R Schmidt H. Leukocyte telomere length is related to brain parenchymal fraction and attention/speed in the elderly: results of the Austrian stroke prevention study. Front Psychiatry. (2020) 11:100. 10.3389/fpsyt.2020.00100 32180739 PMC7059269

[35] Staffaroni AM Tosun D Lin J Elahi FM Casaletto KB Wynn MJ Telomere attrition is associated with declines in medial temporal lobe volume and white matter microstructure in functionally independent older adults. Neurobiol Aging. (2018) 69:68–75. 10.1016/j.neurobiolaging.2018.04.021 29859365 PMC6430612

[36] Deary IJ Ritchie SJ Muñoz Maniega S Cox SR Valdés Hernández MC Luciano M Brain peak width of skeletonized mean diffusivity (PSMD) and cognitive function in later life. Front Psychiatry. (2019) 10:524. 10.3389/fpsyt.2019.00524 31402877 PMC6676305

[37] Rabindran Madanagopal D. Piaget’s theory and stages of cognitive development- an overview. Sch J Appl Med Sci. (2020) 8(9):2152–7. 10.36347/sjams.2020.v08i09.034

[38] Demanelis K Jasmine F Chen LS Chernoff M Tong L Delgado D Determinants of telomere length across human tissues. Science. (2020) 369:6509. 10.1126/SCIENCE.AAZ6876 PMC8108546 32913074

