# Supplementary material for: Telomere length in early childhood and its association with attention: a study in 4–6 year old children
Source: Front Pediatr. 2024 Jun 11;12:1358272. doi: 10.3389/fped.2024.1358272 (PMC11196792; doi:10.3389/fped.2024.1358272)
Supplement: Supplementary file 1 [file Datasheet1.pdf]

Supplementary Material

Supplementary text 1. Detailed information on the CANTAB tests

The children's neurocognitive functioning was assessed using four tasks from the Cambridge Neuropsychological Test Automated Battery (CANTAB) administered on a touch-screen tablet (CANTAB, Cognitive assessment software, 2019). The CANTAB has been shown to provide reliable measurements of executive functions in children as young as four years old. (1) A trained examiner gave instructions for each task according to a standardized protocol provided by the software developers. Instructions were repeated once if the child was unsure or did not understand the task. Each child got the opportunity for a first trial before the actual measurement was done. In total, the child was given four tasks to complete, of which two were designed to assess attention and psychomotor speed, and two were used to measure the child's visual recognition/working memory.

The attention-related tasks included the Motor Screening Task and the Big/Little Circle task. During the Motor Screening Task, the child was shown a series of ten crosses at different locations on the screen, one at a time, and was asked to press on the cross as quickly and accurately as possible. The cross was considered successful if the point of touch was within a close radius around the target's center (based on a screen resolution of 640 × 350 pixels). The average time in milliseconds it took for the child to select the cross successfully was measured during this task, further referred to as response latency, as well as the average pixel units between the touch and the center of the target on successful trials, referred to as error which measured the accuracy of the child. In the Big/Little Circle task, the child was shown two different-sized circles and had to touch the smallest circle as quickly as possible. After 20 displays, the child was instructed to select the largest circle for 20 repetitions. The average time it took to touch the correct stimulus after it was displayed on the screen was assessed, further referred to as response latency.

After evaluating the child's attention and psychomotor speed, we assessed the visual recognition/working memory. In the Spatial Span task, white squares are shown on the screen, of which two briefly change color in a randomized sequence. The child was asked to reproduce the sequence after an auditory cue. If it did so correctly, the sequence was increased by one until the child could not correctly recall it in three consecutive attempts within the same sequence length. The outcome variable for this task was the maximum number of squares the child could remember in the correct sequence, further referred to as span length. The fourth and final task was the

Delayed Matching to Sample task. This task started with displaying a complex visual pattern (sample), which the child had to recognize from four similar patterns that were randomly presented. The patterns consisted of one being identical to the sample pattern, one having the same colors but different shapes, one having the same shape but different colors, and finally, one having different shapes and colors, referred to as the distractor. The selection of patterns to choose from was either presented simultaneously with the sample pattern or after a delay of 0, 4, or 12 seconds after which the sample pattern was not displayed anymore for reference. This task assessed the percentage of a total number of trials upon which the child answered correctly on their first try and the average time it took to select the correct answer on the first try (response latency in milliseconds). Only latencies on the Delayed Matching to Sample task were considered where the child achieved more than 25% correct trials. Additionally, the task evaluated the probability of an error occurring if the previous trial was correctly answered.

Supplementary figure 1. Flowchart for selecting participants in ENVIR*ON*AGE.

912 mother-child pairs were eligible for participation. Due to factors such as loss of contact (145), refusals to participate (321), and language barriers (7), 439 pairs ultimately participated. 239 mother-child pairs provided consent for a blood sample. Based on available biological samples and the exclusion of improper LTL measurements (if all triplicates qPCR ct values showed too strong deviation (ct differences>0.3)), this study included 283 children.

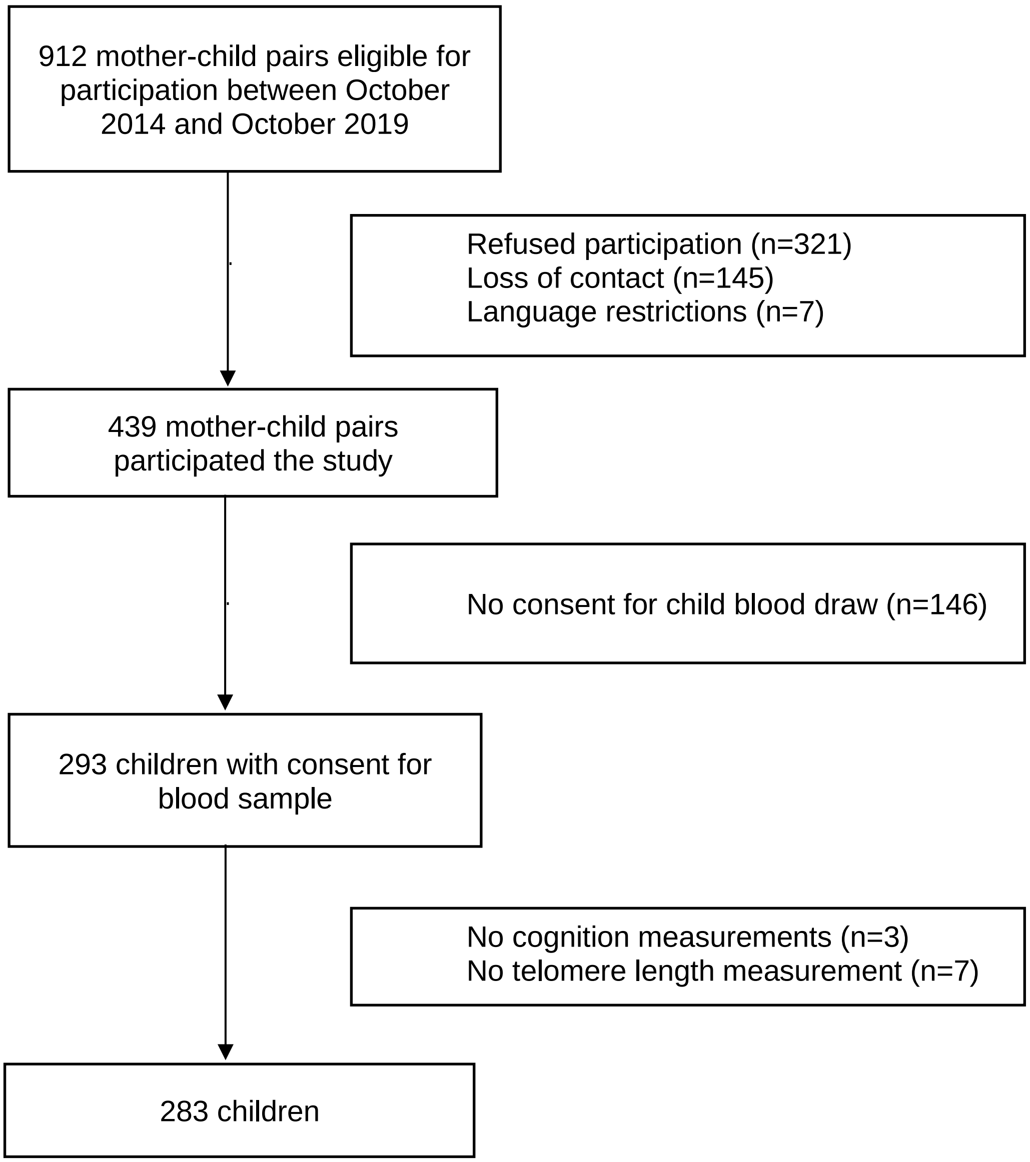

Supplementary text 2. Sample collection and telomere length assay.

Blood samples were obtained from the participating children by venipuncture using a winged steel needle in spray-coated K2EDTA tubes. After collection, the samples were centrifuged at 3200 rpm for 15 min to obtain the buffy coat layer. All samples were stored at -80°C upon analysis. Child leukocyte DNA was extracted using the QIAamp DNA Mini Kit (Qiagen, Inc., Venlo, the Netherlands). DNA quantity and purity was assessed by a Nanodrop 1000 spectrophotometer (Isogen, Life Science, Belgium). DNA was considered pure when the A260/280 was greater than 1.80 and A260/230 was greater than 2.0. DNA integrity was assessed by agarose gel-electrophoresis. DNA samples were normalized to ensure a uniform DNA input of 5 ng for each qPCR. This was checked using the Quant-iT™ PicoGreen® dsDNA Assay Kit (Life Technologies, Europe). All samples were measured in triplicate on a 7900HT Fast RealTime PCR System (Applied Biosystems) in a 384-well format. The telomere-specific qPCR reaction mixture contained 1x QuantiTect SYBR Green PCR master mix (Qiagen, Inc., Venlo, the Netherlands), 2 mM dithiothreitol (DTT), 300 nM telg primer (ACACTAAGGTTTGGGTTTGGGTTTGGGTTTGGGTTAGTG T) and 900 nM telc primer (TGTTAGGTATCCCTATCCCTATCCCTATCCCTATCCCTAACA). Used cycling conditions were: 1 cycle at 95°C for 10 min, 2 cycles at 94°C for 15 sec and 49°C for 2 min, and 30 cycles at 94°C for 15 sec, 62°C for 20 sec, and 74°C for 1 min and 40 sec. The single-copy gene (human β globin) qPCR mixture contained 1x QuantiTect SYBR Green PCR master mix, 400 nM HBG1 primer (GCTTCTGACACAACTGTGTTCACTAGC) and 400 nM HBG2 primer (CACCAACTTCATCCACGTTCACC). Used cycling conditions were: 1 cycle at 95°C for 10 min, 40 cycles at 95°C for 15 sec, and 58°C for 1 min and 20 sec. After each qPCR a melting curve analysis was performed. On each run, a 6-point serial dilution of pooled DNA was run to assess PCR efficiency. 10 inter-run calibrators (IRCs) were run to account for inter-run variability. qPCR curves for each sample were visually inspected and when technical problems were detected or triplicates showed too high variability, samples were removed for further analysis. The reliability of our assay was assessed by calculating the interclass coefficient (ICC) with 95% CI of triplicate measures (T/S ratios, T and S measures separately) using the SPSS statistical package version 25 (IBM Corp. Armonk, NY, USA) based on a mean rating, absolute-agreement, 2-way mixed-effects model. The ICC (95% CI) T/S ratios, telomere runs and single-copy gene runs were 0.976 (0.974 to 0.978), 0.990 (0.988 to 0.991) and 0.978 (0.973 to 0.981), respectively. Based on the 10 IRCs across 16 qPCR plates, the inter-assay ICC was 0.995 (0.987 to 0.999). Relative LTL content was then calculated using the qBase software (Biogazelle, Zwijnaarde, Belgium). In qBase TL was calculated as a calibrated normalized relative quantity (CNRQ). The latter is achieved by first

calculating the RQ based on the delta-Cq method for T, M and S obtained Cq values, using target specific amplification efficiencies. As the choice of a calibrator sample (sample to which subsequent normalization is performed, delta-delta-Cq) strongly influence the error on the final relative quantities (as a result of the measurement error on the calibrator sample), normalization is performed to the arithmetic mean quantification values for all analyzed samples, which results in the NRQ. Finally, as samples are measured over multiple qPCR plates, 9 inter-run calibrators (IRC's) are used to calculate an additional correction factor to eliminate run-to-run differences, resulting into the final T/S ratio (CNRQ). Mathematical calculation formulas to obtain RQ, NRQ and CNRQs are provided by Hellemans et al. (2). Lastly, all TL-values were standardized for sample storage time.

Supplementary table 1. Sensitivity analysis results for the Motor Screening Task Error outcome

		TL	
	n	Estimate (95%CI)	p
Main model excl. disinterest	278	-0.53 (-1.00, --0.06)	**0.029**
Main model + SDQ	256	-0.58 (-1.13, -0.04)	**0.036**
Main model + PSS	283	-0.55 (-1.05,-0.04)	**0.035**
Main model + maternal health condition	283	-0.56 (-1.03, -0,09)	**0.018**
Sex			
Girls	144	-0.49 (-1.05,0.08)	0.09
Boys	139	-0.86 (-1.64,-0.08***)***	***0.03***

Beta (b) estimates are presented as differences in the Motor Screening Task Error outcomes for a 1-IQR increase in TL. The main model is adjusted for the child's age, sex, maternal education, sleephours, BMI of the child, and season of the examinations. We (1) additionally removed cognitive tests if the child was disinterested or not focused to account for response validity (n=5), adjusted for (2) SDQ and (3) PSS results, (4) maternal health conditions including diabetes, preeclampsia, hypertension and infectious diseases, and (4) stratified by sex. Results presented in bold are statistically significant ($p < 0.05$).

References

1. Luciana M. Practitioner review: computerized assessment of neuropsychological function in children: clinical and research applications of the Cambridge Neuropsychological Testing Automated Battery (CANTAB). J Child Psychol Psychiatry Allied Discip. (2003) 44(5):649–63. doi: 10.1111/1469-7610.00152

2. Hellemans J, Mortier G, De Paepe A, Speleman F, Vandesompele J. Qbase relative quantification framework and software for management and automated analysis of real-time quantitative PCR data. Genome Biol. (2008) 8:2. doi: 10.1186/ gb-2007-8-2-r19
